# Association of Permanent Atrial Fibrillation with Cognitive Impairment in Stroke-Censored Patients from Western Romania: A Cross-Sectional Study

**DOI:** 10.3390/diagnostics16091251

**Published:** 2026-04-22

**Authors:** Sergiu-Florin Arnautu, Dragos Catalin Jianu, Minodora Andor, Madalin-Marius Margan, Brenda-Cristiana Bernad, Daniel Rus, Diana-Aurora Arnautu

**Affiliations:** 1 Centre for Cognitive Research in Neuropsychiatric Pathology (Neuro-Psy-Cog), Department of Neurosciences, “Victor Babes” University of Medicine and Pharmacy from Timisoara, E. Murgu Sq., No. 2, 300041 Timisoara, Romania; 2 Department of Internal Medicine, “Victor Babes” University of Medicine and Pharmacy from Timisoara, E. Murgu Sq., No. 2, 300041 Timisoara, Romania; 3 Neurology Clinics I, County Clinical Emergency Hospital, 156 L. Rebreanu Ave., 300736 Timisoara, Romania; 4 Doctoral School, “Victor Babes” University of Medicine and Pharmacy from Timisoara, E. Murgu Sq., No. 2, 300041 Timisoara, Romania; 5 Cardiology Clinics, Timisoara Municipal Clinical Emergency Hospital, Bd. Revolutiei din 1989, No. 12, 300041 Timisoara, Romania; 6 Department of Public Health and Management, “Victor Babes” University of Medicine and Pharmacy from Timisoara, E. Murgu Sq., No. 2, 300041 Timisoara, Romania; 7 Center for Neuropsychology and Behavioral Medicine, “Victor Babes” University of Medicine and Pharmacy from Timisoara, 300041 Timisoara, Romania

**Keywords:** atrial fibrillation, mild cognitive impairment, transcranial Doppler, cerebral hemodynamics, left atrial remodeling

## Abstract

**Background/Objectives**: Cognitive impairment is highly prevalent in atrial fibrillation (AF) and frequently occurs in the absence of overt stroke, implicating non-embolic mechanisms. We hypothesized that atrial remodeling and impaired cerebral hemodynamics are associated with mild cognitive impairment (MCI) in permanent AF. **Methods**: In this cross-sectional study, 252 stroke-free patients with permanent AF receiving direct oral anticoagulants (DOACs) underwent transthoracic echocardiography and transcranial Doppler (TCD) assessment of middle cerebral artery flow. Peak systolic velocity (PSV), end-diastolic velocity (EDV), and resistive index (RI) were analyzed. Multivariable logistic regression identified factors independently associated with MCI, and receiver operating characteristic (ROC) curves evaluated discriminative performance. **Results**: MCI was present in 40% of patients (101/252). AF-MCI patients were older and showed greater left atrial remodeling, reflected by increased left atrial diameter and left atrial volume index (LAVI) (both *p* ≤ 0.001), without differences in left ventricular systolic function. TCD demonstrated reduced EDV and increased RI in the MCI group (all *p* ≤ 0.01), whereas PSV showed minimal differences. In multivariable analysis, age, LAVI, and average RI were independently associated with MCI. Age showed excellent discrimination (AUC 0.858), whereas maximum RI demonstrated moderate discrimination (AUC 0.645; *p* < 0.001 for comparison). **Conclusions**: In stroke-censored permanent atrial fibrillation, cognitive impairment was associated with atrial remodeling and impaired diastolic cerebral perfusion, consistent with a potential contribution of chronic hypoperfusion and increased microvascular resistance. Combined echocardiographic and cerebral hemodynamic assessment may help characterize hemodynamic patterns associated with cognitive impairment in AF.

## 1. Introduction

Dementia affects approximately 5–7% of individuals older than 60 years and remains a major public health challenge, with prevalence projected to nearly triple by 2050 [1,2,3]. Although neurodegeneration is central to its pathogenesis, vascular mechanisms substantially contribute to cognitive decline.

Atrial fibrillation (AF), the most common sustained arrhythmia, is associated with considerable morbidity and mortality and is a well-established risk factor for ischemic stroke [4,5,6]. Increasing evidence, however, demonstrates an independent association between AF and cognitive impairment, even in stroke-free individuals [7,8,9,10,11]. This relationship may be particularly pronounced in younger and middle-aged populations and persists after exclusion of clinically recognized cerebrovascular events [10,11,12,13,14,15].

Beyond overt thromboembolism, proposed mechanisms include chronic cerebral hypoperfusion, beat-to-beat hemodynamic variability, endothelial dysfunction, inflammation, silent microembolization, and progressive small-vessel disease [16,17,18,19,20,21,22]. Nevertheless, the AF–dementia association remains complex due to shared risk factors such as age and cardiometabolic comorbidities, and findings from elderly focused cohorts have been inconsistent [23,24,25,26]. Data integrating direct measures of cerebral hemodynamics with cardiac structural assessment remain limited, particularly in Eastern European populations.

Transcranial Doppler (TCD) enables noninvasive evaluation of cerebral blood flow dynamics, including end-diastolic velocity (EDV) and resistive index (RI), which reflect distal vascular resistance and microvascular function. Echocardiographic assessment of atrial remodeling—especially left atrial (LA) diameter and left atrial volume index (LAVI)—provides structural insight into AF-related cardiomyopathy.

We therefore hypothesized that mild cognitive impairment (MCI) in permanent AF is associated with a distinct cardiac–cerebral hemodynamic phenotype characterized by atrial remodeling and increased cerebrovascular resistance, independent of overt stroke. This study aims to characterize the association between atrial remodeling, cerebral hemodynamic alterations, and cognitive impairment in patients with atrial fibrillation, and to explore imaging markers that may assist in risk stratification without implying causality.

## 2. Materials and Methods

### 2.1. Study Design and Population

This population-based observational cross-sectional study included prospectively and consecutively recruited patients from the cardiology outpatient registry of the Municipal Emergency Hospital in Timișoara, Romania, between January 2024 and December 2025. The study compared clinical, echocardiographic, and cerebral hemodynamic parameters in patients with permanent atrial fibrillation (AF) with and without mild cognitive impairment (MCI). All clinical, echocardiographic, transcranial Doppler, and cognitive assessments were performed at the time of enrollment according to predefined inclusion and exclusion criteria. Transthoracic echocardiography was performed using a GE Vivid S5 (General Electric Healthcare, Chicago, IL, USA) equipped with a 3.4 MHz phased-array transducer for standard two-dimensional imaging. Transcranial color-coded duplex sonography (TCCD) was subsequently performed using a phased-array sector probe (1.5–3.6 MHz, 3S transducer) on the same platform, through standard acoustic windows (transtemporal), with spectral Doppler analysis of the middle cerebral artery. All measurements were obtained by experienced operators, with insonation angles minimized and standardized depth settings applied. Permanent atrial fibrillation (AF) was defined based on a documented clinical diagnosis in the patients’ medical records, characterized by continuous AF accepted by both physician and patient, in accordance with current guideline definitions. To ensure a stable arrhythmic substrate, only patients with a minimum AF duration of ≥1 year were included. Stroke-free status was established through the documented absence of prior stroke or transient ischemic attack (TIA) in medical history, complemented by a neurological clinical evaluation performed at the time of study inclusion. Eligible participants were ≥45 years of age, had a confirmed diagnosis of permanent atrial fibrillation for at least 1 year, were receiving direct oral anticoagulant (DOAC) therapy, and were able to attend outpatient evaluations and complete cognitive testing. The age threshold was selected to focus on middle-aged and older individuals in whom AF-related cognitive changes are more frequently observed.

Stroke-free status was established through the documented absence of prior stroke or transient ischemic attack (TIA) in medical history, complemented by a neurological clinical evaluation performed at the time of study inclusion.

Exclusion criteria included treatment with vitamin K antagonists; contraindications to DOAC therapy (in accordance with current valvular heart disease guidelines, patients with mitral stenosis or mechanical valve were excluded, as DOAC therapy is not recommended in this moderate population); prior stroke or transient ischemic attack; established dementia; major psychiatric illness; neurological disorders affecting cognition; active malignancy; autoimmune disease; advanced hepatic or renal impairment; thyroid dysfunction; hematological disease; significant sensory deficits; or physical limitations precluding cognitive assessment.

Cognitive status was classified using the Mini-Mental State Examination, 2nd edition (MMSE-II). Participants were categorized as having mild cognitive impairment (MCI) if MMSE scores were between 24 and 27, while scores ≥28 were considered cognitively preserved, according to previously published criteria [27].

### 2.2. Study Flow

**Figure 1:**
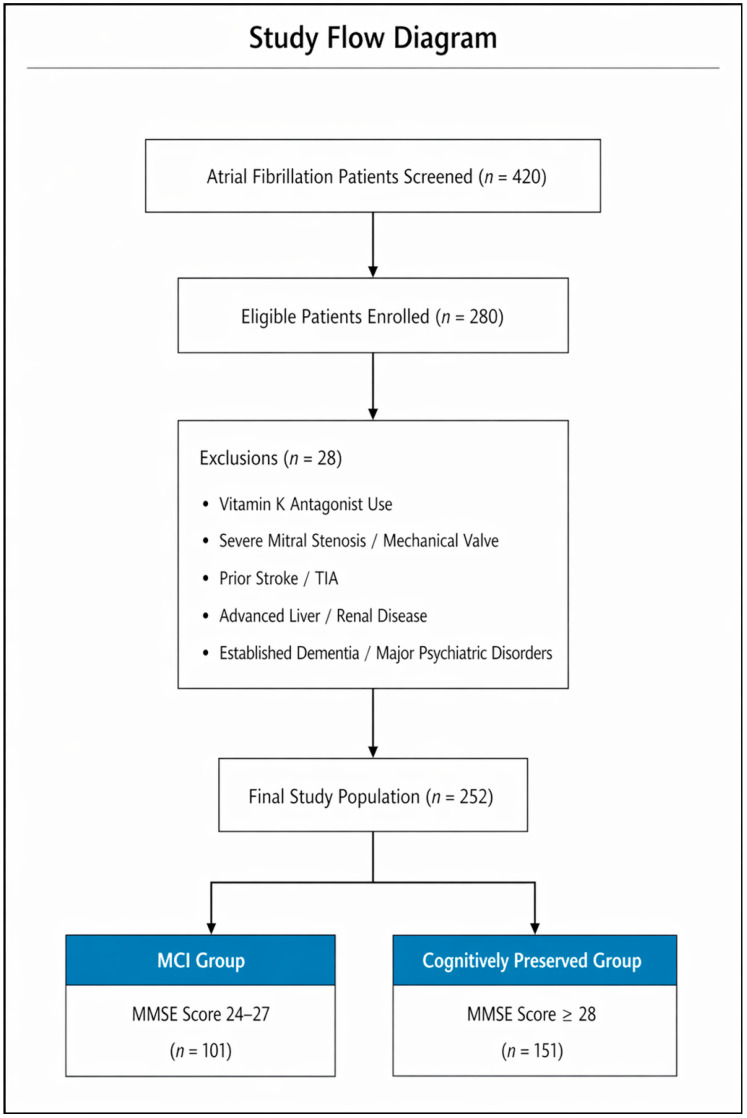
Study flow diagram illustrating patient screening, enrollment, exclusions, and final group allocation according to MMSE-II scores.

The study selection process is summarized in Figure 1. A total of 420 patients with atrial fibrillation were initially screened for eligibility. Of these, 280 met the inclusion criteria and were enrolled. Twenty-eight patients were subsequently excluded due to predefined criteria, including treatment with vitamin K antagonists, contraindications to direct oral anticoagulants, prior stroke or transient ischemic attack, advanced hepatic or renal disease, or established dementia and major psychiatric disorders.

The final study population consisted of 252 patients with permanent atrial fibrillation receiving DOAC therapy. Based on MMSE-II scores, participants were stratified into two groups: 101 patients with mild cognitive impairment (MMSE 24–27) and 151 patients with preserved cognitive function (MMSE ≥ 28).

### 2.3. Ethics Statement

All participants provided written informed consent prior to inclusion. The study was approved by the local Ethics Committee (E-6703/2022) and conducted in accordance with the Declaration of Helsinki. Written informed consent was obtained from all participants prior to study inclusion, as mandated by the Romanian Health Authority. Data were anonymized before analysis.

### 2.4. Clinical and Laboratory Assessment

Data collection included sociodemographic characteristics (age, sex, education, occupational status), cardiovascular risk factors, smoking and alcohol history, and comorbidities (hypertension, coronary artery disease, diabetes mellitus, chronic obstructive pulmonary disease, heart failure, and chronic kidney disease).

Anthropometric measurements included body mass index (BMI). Blood pressure was measured using standardized procedures.

Laboratory parameters were extracted from hospital records and included hemoglobin, platelet count, leukocyte count, lipid profile, C-reactive protein, thyroid hormones, D-dimer, uric acid, blood urea nitrogen, and serum creatinine.

### 2.5. Echocardiographic Assessment

Transthoracic echocardiography was performed using a General Electric Vivid S5 system. Standard parasternal and apical views were obtained according to current guideline recommendations. Left ventricular ejection fraction (LVEF) was calculated using the biplane Simpson method.

Measurements were averaged over 5–7 consecutive cardiac cycles. Interobserver variability was assessed in 20% of randomly selected examinations by two independent cardiologists blinded to clinical data. Intraclass correlation coefficients (ICCs) >0.80 indicated good agreement.

### 2.6. Transcranial Doppler Assessment

Transcranial Doppler ultrasonography was used to assess middle cerebral artery blood flow velocities. Peak systolic velocity (PSV), end-diastolic velocity (EDV), and resistive index (RI) were recorded bilaterally. For analysis, maximum, minimum, and average values were calculated across both sides.

### 2.7. Cognitive Assessment

Cognitive function was evaluated using the Romanian version of the MMSE-II (maximum score 30). Mild cognitive impairment was defined as an MMSE score of 24–27. Participants scoring <24 were excluded. Assessments were conducted by trained personnel under standardized conditions.

### 2.8. Anticoagulation and Risk Scores

DOAC dosing followed current guideline recommendations based on age, renal function, body weight, and concomitant medications. Adherence was assessed through interviews and medical record review.

Thromboembolic risk was estimated using the CHA_2_DS_2_-VASc score, consistent with guideline recommendations at the time of study design. Bleeding risk was assessed using the HAS-BLED score. ATRIA stroke and bleeding risk scores were also calculated.

### 2.9. Clinical Definitions

Hypertension was defined as systolic blood pressure ≥ 140 mmHg and/or diastolic pressure ≥ 90 mmHg or antihypertensive treatment. Diabetes mellitus was defined as HbA1c ≥ 6.5% or glucose-lowering therapy. Chronic kidney disease was defined as eGFR < 60 mL/min/1.73 m^2^ or albuminuria ≥ 30 mg/g.

### 2.10. Statistical Analysis

Continuous variables are presented as mean ± standard deviation or median (interquartile range), as appropriate. Categorical variables are expressed as frequencies and percentages. Normality was assessed using the Shapiro–Wilk test.

Between-group comparisons were performed using Student’s t-test or Mann–Whitney U test for continuous variables and chi-square or Fisher’s exact test for categorical variables.

Correlations were evaluated using Pearson or Spearman coefficients. Multivariable logistic regression was used to identify independent factors associated with MCI. Variables with *p* < 0.10 in univariate analysis were entered into the multivariate model.

Receiver operating characteristic (ROC) curve analysis assessed diagnostic performance. Areas under the curve (AUCs) were compared using the DeLong test. A two-sided *p*-value < 0.05 was considered statistically significant. Analyses were performed using MedCalc^®^ Statistical Software version 23.4.1 (MedCalc Software Ltd., Ostend, Belgium; https://www.medcalc.org; 2025).

No formal a priori sample size calculation was performed, as this was an observational study based on consecutive patient enrollment. The final sample size was determined by the number of eligible participants during the study period. The number of outcome events was sufficient for the multivariable logistic regression analysis according to accepted events-per-variable recommendations.

## 3. Results

### 3.1. Baseline Clinical Characteristics

Baseline clinical characteristics of the study population are summarized in Table 1. A total of 252 patients with atrial fibrillation (AF) were included, comprising 101 patients with mild cognitive impairment (AF-MCI) and 151 patients with normal cognition (AF-NC).

AF-MCI patients were significantly older than AF-NC patients (65.57 ± 5.88 vs. 56.31 ± 6.19 years, *p* < 0.0001). As expected, cognitive scores were significantly lower in the AF-MCI group. MMSE-2 scores were 25.64 ± 4.02 in AF-MCI compared with 27.76 ± 2.61 in AF-NC (*p* < 0.0001).

There were no significant differences between groups regarding sex distribution (male sex: 44% vs. 48%, *p* = 0.53), body mass index (27.6 ± 5.7 vs. 28.4 ± 5.5 kg/m^2^, *p* = 0.26), or prevalence of obesity (*p* = 0.86).

Education level distribution was similar between groups. In the total cohort, low, medium, and high education levels were present in 131 (52%), 66 (26%), and 55 (22%) patients, respectively. In the AF-MCI group the distribution was 90 (50%), 43 (24%), and 47 (26%), while in the AF-NC group it was 41 (57%), 19 (26%), and 12 (17%), with no significant between-group differences (*p* = 0.16).

The prevalence of cardiovascular comorbidities, including hypertension, diabetes mellitus, coronary artery disease, heart failure, peripheral artery disease, and chronic kidney disease, did not differ significantly between groups (all *p* > 0.05). Dyslipidemia (64% vs. 52%, *p* = 0.06) and chronic obstructive pulmonary disease (23% vs. 14%, *p* = 0.06) showed borderline trends toward higher prevalence in the AF-MCI group.

Stroke and bleeding risk scores were comparable between groups. CHA_2_DS_2_-VASc scores were 3.2 ± 1.3 in AF-MCI and 3.4 ± 1.2 in AF-NC (*p* = 0.21), while HAS-BLED scores were 2.22 ± 0.78 and 2.34 ± 0.96, respectively (*p* = 0.27).

The duration of atrial fibrillation was similar between groups (2.7 ± 0.5 vs. 2.6 ± 0.3 years, *p* = 0.07). The distribution of direct oral anticoagulant (DOAC) therapy (dabigatran, rivaroxaban, and apixaban) did not differ significantly between AF-MCI and AF-NC patients (all *p* > 0.05).

Overall, apart from age and cognitive test scores, baseline demographic characteristics, cardiovascular risk factors, AF-related variables, and anticoagulation therapy were comparable between groups.

### 3.2. Baseline Cardiac and Cerebral Hemodynamic Assessment 

Echocardiographic and transcranial Doppler (TCD) parameters of the study population are presented in Table 2.

#### 3.2.1. Echocardiographic Parameters

AF-MCI patients demonstrated significantly greater left atrial (LA) structural remodeling compared with AF-NC patients. LA diameter was significantly larger in the AF-MCI group (4.6 ± 0.52 cm vs. 4.4 ± 0.19 cm, *p* = 0.001). Similarly, left atrial volume index (LAVI) was markedly elevated in AF-MCI patients (46.8 ± 8.4 mL/m^2^ vs. 41.2 ± 9.9 mL/m^2^, *p* < 0.0001).

In contrast, left ventricular parameters did not differ significantly between groups. Left ventricular end-diastolic diameter (LVEDD) was comparable (4.9 ± 0.6 cm vs. 4.8 ± 0.5 cm, *p* = 0.16), as was left ventricular ejection fraction (LVEF) (58.4 ± 4.3% vs. 59.3 ± 5.6%, *p* = 0.15).

#### 3.2.2. Transcranial Doppler Hemodynamic Parameters

Peak Systolic Velocity (PSV): Maximum PSV was lower in AF-MCI patients compared with AF-NC patients (84.23 ± 19.86 cm/s vs. 89.56 ± 22.23 cm/s), reaching borderline statistical significance (*p* = 0.05). Minimum and average PSV did not differ significantly between groups (*p* = 0.21 and *p* = 0.08, respectively).

End-Diastolic Velocity (EDV): All EDV-related parameters were significantly reduced in the AF-MCI group. Maximum EDV was lower in AF-MCI patients (35.11 ± 9.06 cm/s vs. 39.24 ± 12.72 cm/s, *p* = 0.002). Similarly, minimum EDV (28.60 ± 7.83 cm/s vs. 31.54 ± 10.38 cm/s, *p* = 0.01) and average EDV (32.07 ± 7.40 cm/s vs. 35.46 ± 11.15 cm/s, *p* = 0.004) were significantly decreased.

Resistive Index (RI): AF-MCI patients exhibited significantly higher resistive indices compared with AF-NC patients. Maximum RI was elevated in the AF-MCI group (0.62 ± 0.05 vs. 0.59 ± 0.09, *p* = 0.001). Minimum RI (0.56 ± 0.06 vs. 0.54 ± 0.05, *p* = 0.01) and average RI (0.59 ± 0.05 vs. 0.57 ± 0.07, *p* = 0.01) were also significantly increased.

#### 3.2.3. Summary of Hemodynamic Differences

AF patients with mild cognitive impairment (MCI) demonstrated a distinct hemodynamic profile compared with cognitively normal AF patients. The most consistent differences were observed in parameters reflecting atrial remodeling and cerebral diastolic perfusion.

First, AF-MCI patients exhibited significantly greater left atrial structural remodeling, as evidenced by increased LA diameter and LAVI. In contrast, left ventricular size and systolic function (LVEDD and LVEF) did not differ between groups, suggesting that cognitive impairment was not mediated by overt ventricular systolic dysfunction.

Second, transcranial Doppler analysis revealed that diastolic cerebral flow was significantly reduced in AF-MCI patients. All end-diastolic velocity (EDV) parameters were lower in the MCI group, whereas peak systolic velocity (PSV) differences were minimal and largely non-significant. These findings indicate that cerebral perfusion abnormalities in AF-MCI are predominantly diastolic rather than systolic.

Third, resistive index (RI) parameters were consistently elevated in AF-MCI patients, reflecting increased distal cerebrovascular resistance. Maximum and average RI remained independently associated with MCI in multivariate regression analysis. Although RI demonstrated only moderate discriminative capacity in ROC analysis, it contributed independently beyond atrial structural parameters.

Correlation analysis further supported these findings, demonstrating modest but significant associations between cognitive impairment and LA diameter, LAVI, and maximum RI. Age remained the variable most strongly associated with MCI; however, atrial remodeling and cerebrovascular resistance indices also showed independent associations.

Overall, the hemodynamic pattern observed in AF-MCI patients is characterized by increased atrial remodeling, reduced diastolic cerebral flow, elevated cerebrovascular resistance, and preserved left ventricular systolic function.

These findings support a mechanism of cognitive impairment driven by chronic cerebral hypoperfusion and small-vessel dysfunction.

#### 3.2.4. Forest Plot Analysis of the Imagistic Biomarkers Associated with MCI in AF Patients

**Figure 2:**
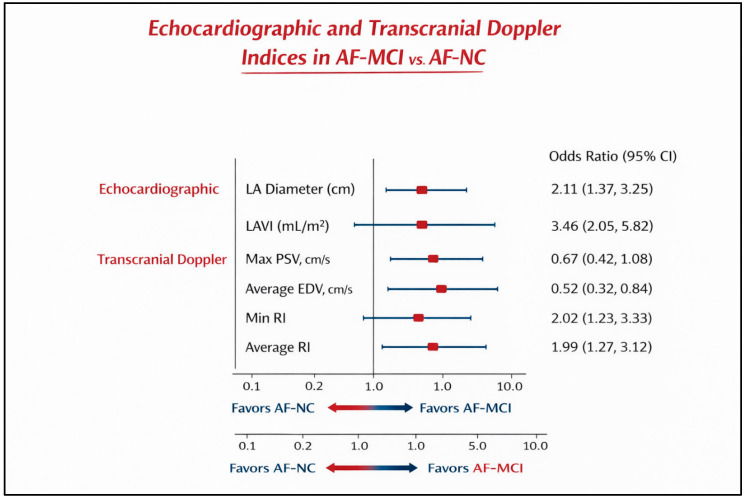
Forest plot showing odds ratios (OR) and 95% confidence intervals (CI) for echocardiographic and transcranial Doppler parameters associated with mild cognitive impairment in patients with atrial fibrillation. Values >1 favor AF-MCI, and values <1 favor AF-NC.

The associations between echocardiographic and transcranial Doppler parameters and the presence of mild cognitive impairment are illustrated in Figure 2 (Forest plot).

As shown in Figure 1, left atrial structural parameters demonstrated strong positive associations with MCI. Increased LA diameter was associated with higher odds of MCI (OR 2.11, 95% CI 1.37–3.25), while LAVI showed the strongest association among all variables (OR 3.46, 95% CI 2.05–5.82).

Among cerebral hemodynamic parameters, reduced average EDV was independently associated with MCI (OR 0.52, 95% CI 0.32–0.84), indicating that lower diastolic cerebral flow was linked to cognitive impairment. Maximum PSV did not reach statistical significance (OR 0.67, 95% CI 0.42–1.08), as the confidence interval crossed unity.

Elevated resistive indices were significantly associated with MCI. Minimum RI (OR 2.02, 95% CI 1.23–3.33) and average RI (OR 1.99, 95% CI 1.27–3.12) both demonstrated independent associations, reinforcing the role of increased cerebrovascular resistance in AF-related cognitive impairment.

Overall, the forest plot demonstrates that atrial remodeling (particularly LAVI) and impaired diastolic cerebral perfusion (reduced EDV and increased RI) are the most robust factors associated with cognitive impairment in AF patients.

### 3.3. Correlation Matrix Analysis

**Figure 3:**
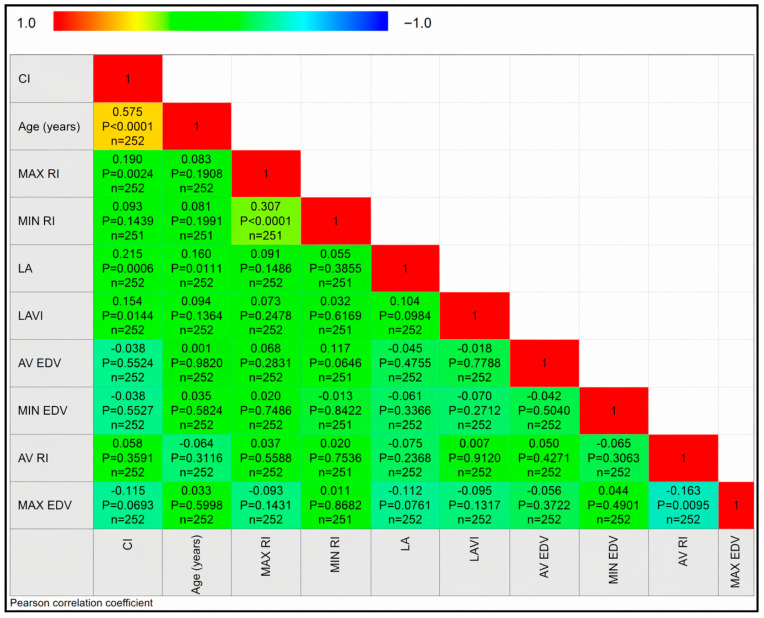
Correlation matrix displaying Spearman correlation coefficients between cognitive impairment (CI), age, echocardiographic indices, and transcranial Doppler parameters in atrial fibrillation patients. Colors represent the strength and direction of correlations.

The correlation matrix illustrating relationships among cognitive impairment (CI), age, echocardiographic parameters, and transcranial Doppler indices is shown in Figure 3.

Age demonstrated a strong positive correlation with CI (r = 0.575, *p* < 0.0001), confirming that advancing age is the dominant factor associated with cognitive impairment in AF patients.

Regarding echocardiographic parameters, LA diameter demonstrated a significant positive correlation with CI (r = 0.215, *p* = 0.0006), while LAVI was also modestly associated with CI (r = 0.154, *p* = 0.0144).

With respect to cerebral hemodynamics, maximum RI was positively correlated with CI (r = 0.1901, 95% CI 0.0681–0.3064, *p* = 0.0024). In contrast, minimum RI was not significantly correlated with CI (r = 0.093, *p* = 0.1439). Average RI showed no significant association (r = 0.058, *p* = 0.3591). No significant correlations were observed between CI and EDV-related parameters (maximum, minimum, or average EDV; all *p* > 0.05).

Inter-parameter correlations revealed strong internal consistency among RI measures, particularly between maximum RI and minimum RI (r = 0.307, *p* < 0.0001), suggesting shared physiological determinants of cerebrovascular resistance.

Overall, the correlation analysis supports age as the primary correlate of CI, with additional contributions from atrial structural remodeling and increased cerebrovascular resistance.

### 3.4. Multivariate Logistic Regression Analysis

To determine independent factors associated with mild cognitive impairment in patients with atrial fibrillation, variables showing statistical significance in univariate comparisons were entered into a multivariable logistic regression model. Education level was evaluated as a potential confounder but was not independently associated with mild cognitive impairment and did not materially influence the effect estimates of the main variables included in the model; therefore, it was not retained in the final multivariable analysis.

The variables included in the multivariable analysis were age, LA diameter, LAVI, maximum EDV, average EDV, maximum RI, minimum RI, and average RI.

After adjustment for potential confounders, age, LAVI, and average RI remained independently associated with the presence of mild cognitive impairment. Increased LAVI was significantly associated with higher odds of MCI (OR 3.46, 95% CI 2.05–5.82, *p* < 0.001). Similarly, elevated average RI independently predicted MCI (OR 1.99, 95% CI 1.27–3.12, *p* = 0.003).

Maximum EDV demonstrated an inverse association with MCI, indicating that reduced diastolic cerebral flow was independently related to cognitive impairment (OR 0.52, 95% CI 0.32–0.84, *p* = 0.007).

In contrast, LA diameter and minimum RI did not retain statistical significance after multivariable adjustment (*p* > 0.05), suggesting that their effects were mediated through atrial remodeling severity and cerebrovascular resistance.

The overall model demonstrated good discrimination for identifying AF patients with MCI (C-statistic > 0.75), indicating acceptable discriminative performance.

### 3.5. ROC Curve Analysis of Independent Factors Associated with MCI

**Figure 4:**
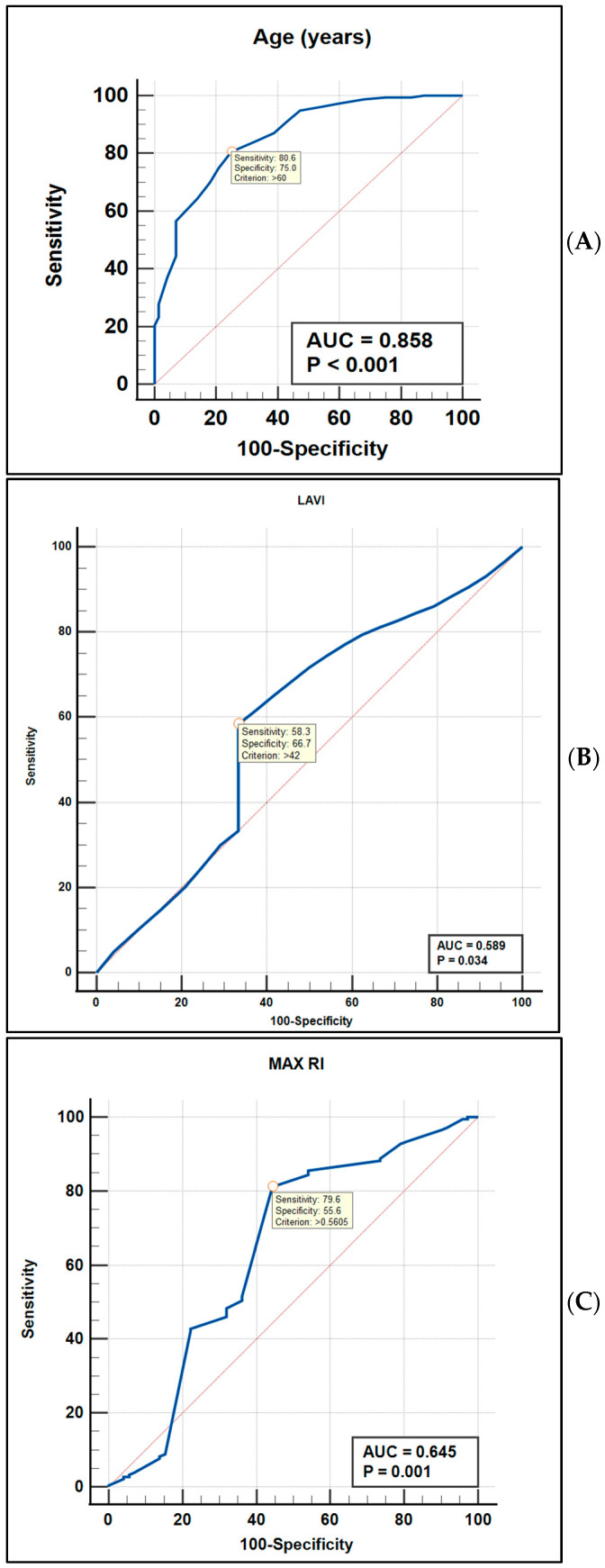

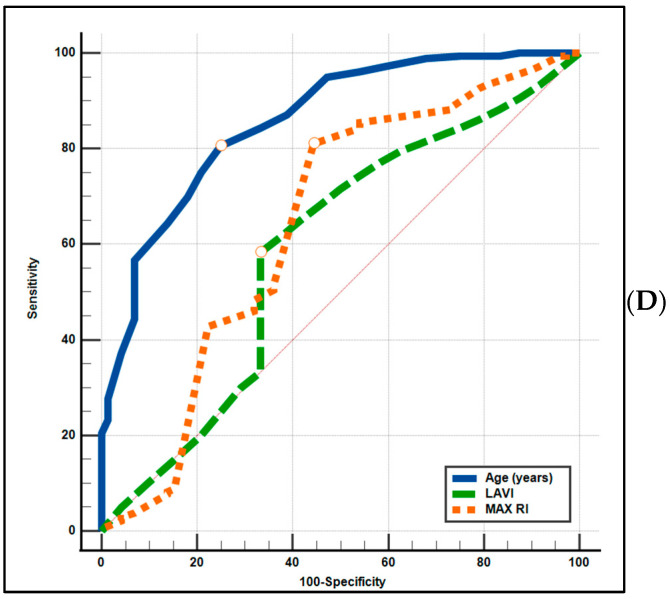
(**A**) ROC curve for age associated with mild cognitive impairment (AUC = 0.858, *p* < 0.001). (**B**) Receiver operating characteristic (ROC) curve evaluating the discriminative performance of left atrial volume index (LAVI) for identifying mild cognitive impairment in patients with permanent atrial fibrillation. The area under the curve (AUC) was 0.589 (*p* = 0.0034), indicating modest diagnostic accuracy. The optimal cutoff value (>42 mL/m^2^) yielded a sensitivity of 58.3% and specificity of 66.7%. (**C**) ROC curve for maximum resistive index (MAX RI) associated with mild cognitive impairment in atrial fibrillation patients (AUC = 0.645, *p* = 0.001). (**D**) Comparative ROC analysis between age, left atrial volume index (LAVI) and maximum resistive index (MAX RI) demonstrating superior discriminative performance of age.

Receiver operating characteristic (ROC) curve analysis was performed to evaluate the discriminative performance of independent factors associated with MCI in AF patients. ROC curves for age, LAVI and maximum resistive index (MAX RI), are shown in Figure 4A, Figure 4B, and Figure 4C, respectively. Their direct comparison is presented in Figure 4D.

MAX RI demonstrated moderate discriminative ability for identifying MCI, with an area under the curve (AUC) of 0.645 (*p* = 0.001). The optimal cutoff value (>0.56) yielded a sensitivity of 79.6% and specificity of 55.6% (Figure 4A). Although statistically significant, the discriminatory performance of MAX RI was modest.

Age showed excellent diagnostic performance, with an AUC of 0.858 (*p* < 0.001). The optimal cutoff value (>60 years) provided a sensitivity of 80.6% and specificity of 75.0% (Figure 4B), indicating strong discriminative capacity for MCI in AF patients.

Direct comparison of ROC curves (Figure 4D) demonstrated that age had significantly superior discriminative performance compared with MAX RI. The difference in AUC between age and MAX RI was 0.214 (95% CI 0.114–0.314, *p* < 0.0001).

Age demonstrated the highest discriminative performance, with the largest area under the curve (AUC 0.858), consistent with excellent diagnostic accuracy. In contrast, MAX RI showed moderate discrimination (AUC 0.645), while LAVI demonstrated modest performance (AUC 0.589). Direct comparison of ROC curves confirmed that age significantly outperformed both MAX RI and LAVI (*p* < 0.0001). Although the incremental diagnostic contribution of MAX RI and LAVI was limited relative to age, their discrimination remained statistically significant and may reflect underlying pathophysiological mechanisms rather than primary screening utility.

Overall, these findings indicate that chronological aging remains the dominant factor associated with MCI in permanent AF, whereas atrial remodeling and cerebrovascular resistance provide complementary, mechanistically informative signals.

## 4. Discussion

### 4.1. Principal Findings

To our knowledge, this is the largest population-based study conducted in Eastern Europe evaluating the association between atrial fibrillation and cognitive impairment.

In this cross-sectional cohort of stroke-free patients with permanent AF receiving DOAC therapy, MCI was common and was associated with a distinct cardiac–cerebral hemodynamic phenotype. MCI was highly prevalent, affecting 40% of patients. This prevalence is consistent with prior epidemiological studies reporting increased rates of cognitive dysfunction in AF populations independent of overt stroke [7,8,9,10,11,16,25], reinforcing the concept that non-embolic mechanisms play a significant role in AF-related cognitive decline.

The principal findings are as follows: (1) MCI was present in 40% of patients despite the absence of prior stroke; (2) patients with MCI exhibited greater left atrial remodeling, reflected by increased LA diameter and LAVI; (3) cerebral hemodynamic abnormalities were predominantly diastolic, characterized by reduced end-diastolic velocity and increased resistive index, whereas systolic flow parameters showed minimal differences; (4) age, LAVI, and RI were independently associated with MCI; (5) age demonstrated the strongest discriminative performance, whereas RI provided additional pathophysiological information.

The observed association between increased left atrial volume index (LAVI) and cognitive impairment is in line with studies identifying atrial cardiomyopathy as a marker of systemic vascular dysfunction and cumulative AF burden [28]. Similar associations between AF and reduced cerebral blood flow or impaired cerebrovascular reactivity have been described in prior studies [18,19,20,29]; however, data integrating echocardiographic markers of atrial remodeling with direct hemodynamic measurements remain limited.

Collectively, these findings support the concept that AF-related cognitive impairment may be linked to non-embolic mechanisms involving atrial structural remodeling and impaired diastolic cerebral perfusion. This interpretation supports emerging evidence that chronic cerebral hypoperfusion and microvascular dysfunction may underlie early cognitive changes in AF [17,29,30]. These findings are further supported by prior observational and mechanistic studies suggesting that atrial structural remodeling and cerebral hemodynamic impairment represent key, interrelated pathways linking atrial fibrillation to cognitive decline, even in the absence of overt cerebrovascular events.

### 4.2. Atrial Remodeling as a Marker of AF-Related Cognitive Vulnerability

Left atrial enlargement and increased LAVI are well-established markers of atrial cardiomyopathy, reflecting chronic pressure and volume overload, atrial fibrosis, and electrical–mechanical remodeling. In our cohort, both parameters were significantly higher in AF patients with MCI, indicating a more advanced stage of atrial disease in cognitively impaired individuals.

This finding is consistent with previous studies demonstrating associations between atrial remodeling and adverse cerebrovascular outcomes, including silent brain infarctions, white matter lesions, and cognitive decline [21,24,28]. Atrial enlargement has been proposed as a surrogate marker of cumulative AF burden and impaired atrial function, both of which may contribute to hemodynamic instability and reduced cerebral perfusion.

Importantly, left ventricular systolic function did not differ between groups in our study, supporting prior observations that AF-related cognitive impairment may occur independently of overt ventricular dysfunction [16,25]. This reinforces the hypothesis that atrial pathology itself—rather than global cardiac failure—plays a central role in the pathogenesis of cognitive decline in AF.

From a mechanistic perspective, atrial remodeling may reflect long-standing exposure to irregular rhythm, impaired atrial contractility, and loss of atrial contribution to ventricular filling, all of which can lead to subtle but chronic reductions in cardiac output and cerebral perfusion. Previous hemodynamic and imaging studies have suggested that such alterations may promote microvascular damage, endothelial dysfunction, and impaired cerebral autoregulation [19,20,29,31].

Taken together, our findings support the growing concept that atrial cardiomyopathy is not only a substrate for thromboembolism but also a marker of systemic vascular and microcirculatory vulnerability contributing to cognitive impairment through non-embolic pathways.

### 4.3. Diastolic Cerebral Hypoperfusion and Increased Microvascular Resistance

Changes in cerebral blood flow and cerebrovascular resistance index represent complementary but mechanistically distinct pathways underlying subclinical brain disease, with accumulating evidence indicating stronger and more consistent associations for cerebrovascular resistance index measures [29]. Moreover, beat-to-beat fluctuations in cerebral blood flow in atrial fibrillation (AF) may increase endothelial shear stress, promoting endothelial dysfunction and progressive microvascular injury [30,31,32].

In our study, the cerebral hemodynamic signature observed in AF patients with mild cognitive impairment (MCI) was predominantly diastolic. Reduced end-diastolic velocity (EDV) and elevated resistive index (RI) indicate increased distal cerebrovascular resistance and impaired diastolic cerebral perfusion. AF-MCI patients exhibited consistently lower EDV values and higher RI values across all parameters, supporting a pattern of impaired downstream perfusion and increased microvascular resistance. These findings are consistent with prior studies demonstrating that AF is associated with impaired cerebrovascular reactivity, increased beat-to-beat variability in cerebral blood flow, and a higher frequency of transient hypoperfusion events [18,19,20,29].

Aging is also associated with increased cerebrovascular resistance and reduced diastolic cerebral blood flow, which may lead to higher RI values and lower EDV even in the absence of AF. In our cohort, age showed the strongest association with cognitive impairment; however, EDV and RI remained associated with MCI after adjustment for age, suggesting that the observed hemodynamic alterations reflect not only physiological aging but also AF-related disturbances in cerebral perfusion.

AF is characterized by an irregular ventricular response and beat-to-beat variability in stroke volume, leading to fluctuations in cerebral perfusion pressure. Chronic exposure to such hemodynamic instability may impair cerebral autoregulation, promote endothelial dysfunction, and contribute to progressive microvascular remodeling. In addition, the loss of coordinated atrial contraction (“atrial kick”) reduces late diastolic ventricular filling, potentially lowering effective stroke volume and further amplifying variability in cerebral blood flow. These mechanisms may propagate to the cerebral circulation, where irregular perfusion dynamics favor distal hypoperfusion [20,21,22,33].

Notably, peak systolic velocity was largely preserved in our cohort, reinforcing the concept that AF-related cognitive impairment is more closely related to sustained microvascular and diastolic dysfunction than to large-vessel systolic flow impairment. This pattern aligns with mechanistic models of chronic cerebral hypoperfusion and subclinical white matter injury. Consistent with this interpretation, prior neuroimaging studies have demonstrated an increased burden of white matter hyperintensities and silent cerebral infarctions in AF patients, supporting the presence of chronic microvascular damage [21,24].

From a clinical perspective, these findings suggest that transcranial Doppler–derived indices such as resistive index may serve as accessible, non-invasive markers of microvascular dysfunction, potentially enabling earlier identification of AF patients at increased risk of cognitive decline.

Overall, these findings support a pathophysiological framework in which cognitive impairment in AF arises from the interaction between age-related vascular changes, atrial structural disease, and chronic hemodynamic instability, ultimately leading to impaired microvascular perfusion rather than overt large-vessel pathology.

### 4.4. Age Versus Hemodynamic Biomarkers: Dominant and Incremental Effects

In population-based studies, aging is the strongest factor associated with cognitive decline, and the relationship between atrial fibrillation and cognitive impairment becomes more complex with advancing age. In our cohort of AF patients aged ≥45 years, age remained the variable most strongly associated with MCI, consistent with previous epidemiological data.

Aging is also associated with structural and functional changes in the cerebral vasculature, including increased arterial stiffness, impaired endothelial function, and reduced microvascular compliance, all of which may contribute to higher resistive index values and reduced diastolic cerebral flow. These physiological changes may partially explain the association between age and cognitive impairment observed in our study.

However, beyond chronological aging, we identified a distinct cardiac–cerebral hemodynamic profile characterized by greater atrial remodeling and increased cerebrovascular resistance. In the multivariable analysis, LAVI and RI remained associated with MCI after adjustment for age, suggesting that AF-related structural and hemodynamic alterations may contribute independently of aging.

These findings support a model in which age represents the dominant background factor for cognitive decline, whereas atrial remodeling and increased cerebrovascular resistance may provide additional mechanistic information regarding AF-related vulnerability. In this context, echocardiographic and transcranial Doppler parameters may help to characterize the hemodynamic phenotype associated with cognitive impairment rather than to establish causal relationships.

In our cohort of AF patients aged ≥45 years, age >60 years showed the strongest independent association with MCI. However, beyond chronological aging, we identified a distinct cardiac–cerebral hemodynamic phenotype characterized by atrial remodeling and increased cerebrovascular resistance, independent of overt stroke and preserved left ventricular systolic function. By integrating echocardiographic structural markers with transcranial Doppler–derived indices of cerebral perfusion, our findings provide mechanistic support for a chronic hypoperfusion and microvascular dysfunction pathway linking AF to early cognitive vulnerability. Although age demonstrated superior discriminative performance, resistive index may retain clinical value as a mechanistic biomarker to refine cognitive risk stratification in AF. Even if AF duration was relatively short and comparable between groups, its potential cumulative impact on cerebral perfusion and cognition cannot be excluded and warrants longitudinal investigation.

In clinical practice, a TCD-derived resistance index could help identify AF patients in whom microvascular mechanisms are more prominent and for whom targeted vascular risk modification and longitudinal cognitive surveillance may be warranted. In clinical terms, age identifies risk, whereas RI and LAVI may help define mechanism.

Previous studies have identified additional clinical factors associated with cognitive decline in AF, including diabetes mellitus and female sex, as reported in observational cohorts of newly diagnosed non-valvular AF patients as well as in large population-based studies [4,9]. For example, an observational study in newly diagnosed non-valvular AF patients found that metabolic factors and female sex were significantly associated with dementia [4]. In our cohort, these variables were not significantly associated with mild cognitive impairment. Several differences in study design may explain these discrepancies. First, our study excluded patients with prior stroke and focused on mild cognitive impairment rather than established dementia, which may reduce the influence of long-term vascular risk factors. Second, all patients were receiving direct oral anticoagulant therapy, which may attenuate the impact of thromboembolic and metabolic mechanisms. Third, our population was relatively younger and consisted of consecutively evaluated outpatients, which may limit the effect of comorbidities that become more relevant in older or higher-risk cohorts. These differences suggest that the relative contribution of clinical risk factors and hemodynamic mechanisms to cognitive impairment in AF may vary across populations and study settings.

### 4.5. Clinical Implications

Our results support a conceptual model in which AF-associated cognitive impairment reflects not only embolic risk but also chronic hemodynamic and microvascular injury. From a translational perspective, echocardiographic assessment of atrial remodeling may serve as a structural marker of cognitive risk; TCD-derived RI may function as a no-invasive surrogate of cerebrovascular resistance; combined cardiac–cerebral phenotyping may refine risk stratification beyond traditional clinical scores.

Such multimodal evaluation could identify AF patients who may benefit from intensified vascular risk modification, rhythm control strategies, or longitudinal cognitive surveillance.

### 4.6. Limitations

This study has several limitations. The cross-sectional design precludes causal inference. Cognitive classification relied on the MMSE-II, which, although widely used, has limited sensitivity for detecting executive dysfunction and vascular cognitive impairment compared with the Montreal Cognitive Assessment. The choice of MMSE-II was based on its widespread clinical use, the availability of validated normative data in the Romanian population, and its feasibility in the outpatient setting. However, the use of fixed score cutoffs may introduce misclassification bias, particularly in relation to education level and premorbid cognitive reserve. Although education level was included as a covariate in the analysis, residual confounding related to cognitive reserve cannot be completely excluded. Transcranial Doppler measures flow velocity rather than absolute cerebral blood flow and is operator-dependent. Brain MRI was not systematically performed in this cohort, and neuroimaging data were not available for standardized analysis, limiting structural correlation with hemodynamic findings. Silent cerebral infarctions could not be systematically excluded due to the absence of routine neuroimaging, and may have contributed to cognitive impairment. Other potential sources of cardioembolism, such as mitral annular calcification or significant aortic valve disease, were not systematically assessed and may represent residual confounders. Advanced echocardiographic parameters, including left ventricular global longitudinal strain, left atrial strain, and indices of diastolic function and filling pressures, were not assessed. This limits the detection of subclinical systolic and atrial dysfunction and represents an important limitation of the study. Residual confounding, including AF burden quantification and blood pressure variability, cannot be excluded.

### 4.7. Future Directions

Prospective longitudinal studies are required to determine whether atrial remodeling and elevated RI predict incident cognitive decline. Integration of brain MRI, AF burden assessment, and cerebral autoregulation testing would further delineate hypoperfusion versus micro-embolic pathways. Interventional studies evaluating whether rhythm control or vascular optimization modifies cerebral hemodynamic parameters and cognitive trajectories would be of high translational relevance.

## 5. Conclusions

In stroke-free patients with permanent atrial fibrillation receiving DOAC therapy, mild cognitive impairment was associated with greater left atrial remodeling and with a cerebral hemodynamic profile characterized by reduced diastolic flow velocity and increased cerebrovascular resistance. Age showed the strongest association with MCI, whereas resistive index demonstrated moderate discriminative performance and provided additional pathophysiological information. These findings are consistent with a potential contribution of chronic hypoperfusion and increased microvascular resistance to cognitive impairment in atrial fibrillation and suggest that the combined use of echocardiographic and transcranial Doppler parameters may help characterize the hemodynamic profile associated with cognitive dysfunction in AF.

## Figures and Tables

**Table 1:** Baseline clinical characteristics of the patients with atrial fibrillation.

Total	Total AF 252	AF-MCI *n* = 101	AF-NC *n* = 151	*p* Value
Age (years)	62.92 ± 72 (45–83)	65.57 ± 5.88 (49–83)	56.31 ± 6.19 (45–70)	<0.0001
MMSE-2 (X ± SD)	26.71±	3.52.64 ± 4.02	27.76 ± 2.61	<0.0001
Male sex, *n* (%)	116 (46)	44 (44)	72 (48)	0.53
Education degree High school College University	131 (52) 66 (26) 55 (22)	90 (50) 43 (24) 47 (26)	41 (57) 19 (26) 12 (17)	0.16 0.73 0.13
Body mass index, kg/m^2^	28.8 ± 5.6	27.6 ± 5.7	28.4 ± 5.5	0.26
Obesity	85 (33)	33 (32)	50 (33)	0.86
Hypertension, *n* (%)	181 (72)	77 (77)	104 (69)	0.16
Diabetes mellitus, *n* (%)	70 (28)	29 (29)	41 (27)	0.73
Dyslipidemia, *n* (%)	143 (57)	64 (64)	79 (52)	0.06
Current smoker, *n* (%)	29 (12)	14 (14)	15 (10)	0.33
Coronary artery disease, *n* (%)	73 (29)	31 (31)	42 (28)	0.60
COPD	44 (20)	23 (23)	21 (14)	0.06
Heart failure, *n* (%)	76 (30)	34 (34)	42 (28)	0.31
Peripheral artery disease	17 (7)	8 (8)	9 (6)	0.53
Chronic kidney disease *n* (%)	64 (25)	28 (28)	36 (24)	0.47
CHA_2_DS_2_-VAsc score	3.3 ± 1.2	3.2 ± 1.3	3.4 ± 1.2	0.21
HAS-BLED score	2.22 ± 0.78	2.22 ± 0.78	2.34 ± 0.96	0.27
Duration of AF, years (X ± SD)	2.6 ± 0.4	2.7 ±0.5	2.6 ± 0.3	0.07
eGFR, mL/min/1.73 m^2^	74.0 ± 18.9	75.3 ± 18.4	73.1 ± 19.2	0.36
Hematocrit, %	42.5 ± 4.4	42.4 ± 4.7	41.7 ± 4.3	0.22
Hemoglobin, g/dL	13.2 ± 4.2	13.4 ± 4.5	13.1 ± 4.1	0.58
Hemoglobin A1c, %	5.7 ± 1.9	5.9 ± 1.5	5.7 ± 2.2	0.39
Total cholesterol, mg/dL	223 ± 32	225 ± 34	223 ± 30	0.62
Type of DOAC, *n* (%) Dabigatran Rivaroxaban Apixaban	38 (15) 25 (10) 189 (75)	12 (12) 8 (8) 72 (80)	26 (17) 17 (11) 109 (72)	0.27 0.58 0.15

Notes: Data are presented as mean ± SD, or number (%). Abbreviations: AF, Atrial Fibrillation; NC, Normal Cognition; MCI, Mild Cognitive Impairment; MMSE-2, MMSE, Mini-Mental State Examination; MoCA, Montreal Cognitive Assessment; COPD, chronic obstructive pulmonary disease; CHA2DS2-VA, score to estimate thromboembolic risk in patients with atrial fibrillation.; HAS-BLED, score to estimate the risk of major bleeding in patients with atrial fibrillation receiving anticoagulant therapy; DOAC, direct oral anticoagulant therapy.

**Table 2:** Baseline Echocardiographic and Transcranial Doppler Indices.

Total	Total AF 252	AF-MCI *n* = 101	AF-NC *n* = 151	*p* Value
LA diameter (cm)	4.5 ± 0.25	4.6 ± 0.52	4.4 ± 0.19	0.001
LAVI (mL/m^2^)	43.3 ± 9.7	46.8 ± 8.4	41.2 ± 9.9	<0.0001
LVEDD (cm)	4.8 ± 0.5	4.9 ± 0.6	4.8 ± 0.5	0.16
LVEF (%)	59.3 ± 5.1	58.4 ± 4.3	59.3 ± 5.6	0.15
Maximum PSV (x ± SD)	87.43 ± 21.51	84.23 ± 19.86	89.56 ± 22.23	0.05
Minimum PSV (x ± SD)	73.7 ± 19.05	71.88 ± 18.71	74.92 ± 19.24	0.21
Average PSV (x ± SD)	80.61 ± 19.37	78.05 ± 18.53	82.31 ± 19.76	0.08
Maximum EDV (x ± SD)	37.18 ± 11.31	35.11 ± 9.06	39.24 ± 12.72	0.002
Minimum EDV (x ± SD)	30.07 ± 9.36	28.60 ± 7.83	31.54 ± 10.38	0.01
Average EDV (x ± SD)	34.10 ± 9.95	32.07 ± 7.40	35.46 ± 11.15	0.004
Maximum RI (x ± SD)	0.60 ± 0.08	0.62 ± 0.05	0.59 ± 0.09	0.001
Minimum RI (x ± SD)	0.55 ± 0.06	0.56 ± 0.06	0.54 ± 0.05	0.01
Average RI (x ± SD)	0.57 ± 0.06	0.59 ± 0.05	0.57 ± 0.07	0.01

Notes: Maximum refers to the maximum value of both sides, minimum refers to the minimum value of both sides, average refers to the average value of both sides (e.g., “maximum PSV” means the maximum value of the peak systolic blood flow velocity in either left middle cerebral artery or right middle cerebral artery). Abbreviations: AF, Atrial Fibrillation; NC, Normal Cognition; MCI, Mild Cognitive Impairment; PSV, Peak Systolic Velocity of blood flow in middle cerebral artery; EDV, End Diastolic Velocity of blood flow in middle cerebral artery; RI, Resistive Index; PI, Pulsatility Index.

## Data Availability

The raw data supporting the conclusions of this article will be made available by the first author, SFA, on request.

## Abbreviations

The following abbreviations are used in this manuscript:

AF	Atrial Fibrillation
CI	Cognitive Impairment
DOAC	Direct Oral Anticoagulant
EDV	End Diastolic Velocity of Blood Flow in Middle Cerebral Artery
LA	Left Atrium
LAVI	Left Atrial Volume Index
LVEDD	Left Ventricular End Diastolic Diameter
LVEF	Left Ventricular Ejection Fraction
MAX	Maximal
MCI	Mild Cognitive Impairment
MoCA	Montreal Cognitive Assessment
NC	Normal Cognition
PSV	Peak Systolic Velocity of Blood Flow in Middle Cerebral Artery
RI	Resistive Index
SD	Standard Deviation
